# Effects of Elastic Couplings in a Compressed Plate Element with Cut-Out

**DOI:** 10.3390/ma15217752

**Published:** 2022-11-03

**Authors:** Katarzyna Falkowicz, Sylwester Samborski, Paolo Sebastiano Valvo

**Affiliations:** 1 Faculty of Mechanical Engineering, Department of Machine Design and Mechatronics, Lublin University of Technology, Nadbystrzycka 36, 20-618 Lublin, Poland; 2 Faculty of Mechanical Engineering, Department of Mechanics, Lublin University of Technology, Nadbystrzycka 36, 20-618 Lublin, Poland; 3 Department of Civil and Industrial Engineering, University of Pisa, Largo Lucio Lazzarino, I-56122 Pisa, Italy

**Keywords:** extension–twisting coupling, extension–bending coupling, FEM, plates elements, coupled laminates, asymmetrical laminates, elastic element

## Abstract

Analytical calculations were performed on carbon fiber-reinforced polymer (CFRP) laminates in an asymmetrical configuration. The asymmetric configuration of composites was investigated, where extension–twisting and extension–bending couplings were used to obtain the elastic element. Analysis of the presence of elastic couplings was conducted according to Classical Laminate Theory (CLT). Components of matrices A, B, and D, as well as the parameters *D*_c_ and *B*_t_, were obtained using the MATLAB software environment. The results show that couplings between the extension and bending, as well as between the extension and twisting, were strongly dependent on specimen plies’ orientation. Moreover, additional analysis was performed on the influence of layer angle on the terms which are components of the *B*_t_ and *D*_c_ coefficients. The results indicate that the angle of laying fibers around 45–50° significantly amplifies the effects of elastic couplings.

## 1. Introduction

Composite materials, thanks to their favorable properties, are used in many kind of industries such as aircraft and architecture [1,2,3,4]. The growing use of these materials makes it necessary to conduct extensive research on structures made of these materials under complex loads. An important issue is the load capacity of composite structures because thin-walled elements, due to their characteristics, in certain cases, can lose stability under acceptable operating loads [5,6,7]. For that reason, apart from strength requirements, thin-walled structures must also satisfy specified requirements for stiffness to prevent the structure from premature failure due to a loss of stability by its elements. These issue have been discussed in many research works [8,9,10].

Recently, more attention has been focused on composites in an asymmetrical configuration. Laminates with complex mechanical couplings can find applications not only in the aerospace sector, with which they have been traditionally associated, but they also have potential, for example, as an enabling technology in large offshore wind turbine blades. York [11,12] studied some unexplored laminate design area containing different kinds of mechanical coupling, which includes all interactions between extension, bending, shearing, and twisting.

The analyses in this area may help to raise interest in the potential for exploiting mechanically coupled materials, particularly from a manufacturing perspective. Applications of the unique couplings inherent in asymmetric laminates can provide design advantages.

However, these kinds of laminated materials also present a challenge in the design process, since couplings occurring in asymmetrical configurations require more careful analysis than traditional isotropic and orthotropic plates.

Specifically, it is important to understand the behavior of these laminates in compressive loading and their buckling and postbuckling behavior since many structural components undergo significant compressive loads. Leissa [13] made a comprehensive summary of the state of the art in buckling and postbuckling of composite structures. She reviewed almost 400 works performed in the past few decades. Most of these works relate to symmetric and balanced composite plates, but some work has been conducted on asymmetric laminates. It has been confirmed that the elastic couplings in laminates reduce the structural stiffness, resulting in lower buckling loads and more compliant behavior. Moreover, Leissa showed [14] that many buckling results were presented on the false assumption that bifurcation buckling can occur, when in fact simply supported rectangular plates consisting of cross-ply laminates with extension–bending coupling will bend, and not buckle, when subjected to in-plane compressive load. However, those works have been limited to the linear buckling problem of laminates. Some examples of analytical work on the nonlinear analysis of plates, with applications of asymmetric composite plates, were assembled by Chia [15]. Detailed descriptions of stiffness matrix couplings for asymmetric laminates are presented by York [12], Altenbach [16], and Samborski [17,18].

The above analyses confirm that an effective way to design laminate and its properties is by the design of the laminate stacking sequences. A suitable ply design can not only endow laminates with a special coupling effect [19,20], but can also improve the mechanical properties of laminates [21]. Thus, a thorough understanding of the compressive response of these laminates is necessary.

In this paper, we present an analysis of the behavior of thin-walled plates weakened by cut-outs in asymmetrical configurations of composites where extension–twisting and extension–bending couplings were used. The main aim of the work was to design a specimen with a maximized B-T coupling. Additional analytical modeling was conducted to gauge the influence of the angle of layers on the values of the *D*_c_ and *B*_t_ coefficients, where *B_t_* is responsible for B-T coupling intensity. Moreover, the effects of matrix components which influence *B*_t_ and *D*_c_ coefficients were investigated. The motivation for the research presented here was the need to recognize the effect of mechanical coupling in plate elements which can work as elastic elements.

## 2. Mechanical Couplings

According to Classical Laminate Theory (CLT), the laminate constitutive equations have the following form [22,23,24,25,26]:

(1) $$\left\{\begin{array}{c} \left\{N\right\}\\ \left\{M\right\} \end{array}\right\}=\left[\begin{array}{cc} \left[A\right] & \left[B\right]\\ \left[B\right] & \left[D\right] \end{array}\right]\left\{\begin{array}{c} \left\{\varepsilon \right\}\\ \left\{\kappa \right\} \end{array}\right\}$$

where *N* is the normal forces matrix and *M* is the bending moments matrix. ε and κ are the mid-plane strain and curvatures of the laminate, respectively, A, B, and D are the stiffness matrices determined according to the following expressions:

- Extension stiffness matrix:

(2) $$A_{ij}=\overset{N}{\underset{k=1}{\sum }}{\left({\bar{Q}}_{ij}\right)}_{k}\left(z_{k}-z_{k-1}\right)$$

- Coupling stiffness matrix:

(3) $$B_{ij}=\frac{1}{2}\overset{N}{\underset{k=1}{\sum }}{\left({\bar{Q}}_{ij}\right)}_{k}\left(z_{k}^{2}-z_{k-1}^{2}\right)$$

- Bending stiffness matrix:

(4) $$D_{ij}=\frac{1}{3}\overset{N}{\underset{k=1}{\sum }}{\left({\bar{Q}}_{ij}\right)}_{k}\left(z_{k}^{3}-z_{k-1}^{3}\right)$$

where *Q_ij_* is the transformed reduced stiffness (*i*, *j* = 1, 2, 6) and *z_k_* is the layer k interface distance from the laminate mid-plane.

Moreover,

A_ij_ = A_ji,_ B_ij_ = B_ji,_ D_ij_ = D_ji_ (5)

The coupling stiffness matrix [B] shows coupled relationships between the components of the load and deformation state. Thus, in the general case of a laminate, target states are also accompanied by flexural states (i.e., bending and torsion), and vice versa.

The above stiffness submatrices have the following forms:

Extension stiffness matrix:

(6) $$\left[A\right]=\left[\begin{array}{ccc} A_{11} & A_{12} & A_{16}\\ A_{21} & A_{22} & A_{26}\\ A_{61} & A_{62} & A_{66} \end{array}\right]$$

Coupling stiffness matrix:

(7) $$\left[B\right]=\left[\begin{array}{ccc} B_{11} & B_{12} & B_{16}\\ B_{21} & B_{22} & B_{26}\\ B_{61} & B_{62} & B_{66} \end{array}\right]$$

Bending stiffness matrix:

(8) $$\left[D\right]=\left[\begin{array}{ccc} D_{11} & D_{12} & D_{16}\\ D_{21} & D_{22} & D_{26}\\ D_{61} & D_{62} & D_{66} \end{array}\right]$$

Asymmetric laminates are characterized by the occurrence of additional asymmetric couplings when particular terms of the coupling stiffness matrix are equal to B_11_ ≠ 0, B_12_ ≠ 0, and B_22_ ≠ 0 (normal loads–flexural loads) or B_66_ ≠ 0 (shearing load–torsional strain) or B_16_ ≠ 0 and B_26_ ≠ 0 (normal load–torsional strain). Each of the matrices with the “0” index consists of zeros. The B coupling matrix, besides the B_0_ variant, can additionally have five different forms of couplings: B_L_, B_T_, B_LT_, B_S_, and B_F_, which were described in detail by York [11,12,27]. It is worth mentioning here that the coupled forms can be described in terms of the response the laminate has to various combinations of force and moment resultants, using a cause and effect relationship. For example, laminate is described as an E–B laminate if extension (E) causes a bending (B) effect, whereas if shear causes an extension effect, then the laminate is described as an E-S laminate. Moreover, each cause and effect relationship is reversible. Thanks to these properties, it is possible to shape the mechanical properties of composite elements by designing the required couplings of strain states. This conception was employed in the present study to find solutions for changing the lowest form of buckling for higher flexural–torsional form to obtain the elastic element. According to the subscript identification method of composite laminate stiffness matrices proposed by the ESDU (1994) [28], the laminates are classified and identified.

## 3. Research Subject

**Figure 1:**
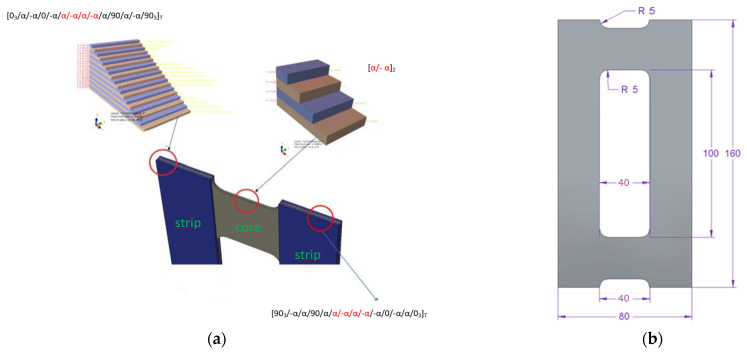
(**a**) Scheme of tested element for angle α = 45°; (**b**) scheme of specimen with the most important parameters.

The main aim of this work was to design a specimen with maximized B-T coupling and determine how we can influence this coupling intensity. The tested structure consists of two groups of laminates (core and vertical strips), which can be called sublaminates—this term is widely used in the literature (e.g., in [18,25,28], where the authors analyze the influence of couplings, e.g., in the DCB sample, treating both branches/legs of the sample as separate sublaminates). It follows that for sublaminates, treated as separate laminates, it is justified to use the description of mechanical couplings using the ABD matrix. Moreover, there are additional parameters describing the intensity of the couplings, such as *B*_t_ and *D*_c_ [25,29], which we took into account when selecting the type of couplings. The calculations of this parameter were performed in MATLAB (together with the determination of the components of the remaining matrices), separately for the core and strips, as well as for the entire plate laminate, the “final laminate configuration”. Additionally, the determined coupling matrices were compared with the matrix representations presented in York [12]. Figure 1a presents a model of a plate element with a separate core and vertical strips and Figure 1b presents a specimen with geometric parameters and a coordinate system.

The current study was focused on the ply sequences expected to have the strongest effect on obtaining the flexural–torsional buckling mode as the naturally lowest mode of buckling. For this reason, extension–twisting (E-T) and extension–bending (E-B) coupled laminate classes were chosen for consideration. Table 1 presents the analyzed configuration.

It is worth noting that the minimization of one coupling effect can increase another, and for this reason, two additional parameters, mentioned above, were introduced by some authors [29]. The first parameter, *D*_c_, is a non-dimensional bending stiffness ratio, defined as a function of the D matrix terms:

(9) $$D_{c}=\frac{D_{12}^{2}}{D_{11}D_{22}}$$

Note that small values of *D*_c_ occur in the plane stress state (pσ), as its physical sense is

(10) $$D_{c}=1-\frac{{\left(EJ\right)}_{p\sigma }^{}}{{\left(EJ\right)}_{p\varepsilon }}$$

where *pε* stands for the plane strain state. Equation (10) clarifies the parameter name used above and leads to the conclusion that the minimization of *D*_c_ causes a decrease in computation errors, as the plies are assumed to experience the plane stress (*pσ*), and as such, they are in fact specially orthotropic layers. In the case of laminates rich in 0° plies, the *D*_c_ is always small.

The second parameter, *B*_t_, is a measure of the B-T coupling intensity:

(11) $$B_{t}=\frac{\left|D_{16}\right|}{D_{11}}$$

## 4. Constitutive Relations

Equations (12)–(15) show the results of analytical calculations of matrix components for the tested configuration for a fiber angle of α = 45°. The formulas that were used for the calculations can be found in publicly available works [16,19,24]. The range of tested angles was 0° to 90°. The constitutive relations are useful to confirm the precisely matched coupling stiffness properties and for gaining qualitative insight into the coupling terms.

The engineering constants of the ply material were carbon fiber/epoxy (EP137-CR527/100-35, where resin content by weight equals 35 ± 3, reinforcement: 100 g/m^2^ IM), and they were experimentally determined according to the ISO standard: *E*_1_ = 143.53 GPa, *E*_2_ = 5.83 Gpa, *G*_12_ = 3.85 Gpa, and *ν*_12_ = 0.36. The lamina thickness was *t* = 0.105 mm, giving an 18-ply laminate thickness *H* = 1.89 mm for the PN1 configuration.

### Laminate 1

The constitutive matrix for the core representing E-T and S-B couplings is given in Equation (12):

(12) $$\left[\begin{array}{cc} \begin{array}{ccc} 17822.99 & 1459.19 & 0\\ 14593.19 & 17822.99 & 0\\ \begin{array}{c} 0\\ \cdots \end{array} & \begin{array}{c} 0\\ \cdots \end{array} & \begin{array}{c} 15322.54\\ \cdots \end{array} \end{array} & \begin{array}{ccc} \begin{array}{cc} \vdots & 0 \end{array}\; & 0 & -763.11\\ \begin{array}{cc} \vdots & 0 \end{array}\; & 0 & -763.11\\ \begin{array}{c} \begin{array}{cc} \vdots & -763.11 \end{array}\\ \;\begin{array}{cc} \cdots & \;\cdots \end{array} \end{array} & \begin{array}{c} -763.11\\ \cdots \end{array} & \begin{array}{c} 0\\ \cdots \end{array} \end{array}\\ symmetry & \begin{array}{ccc} \begin{array}{cc} \vdots & 262 \end{array}\; & 214.52 & \;0\\ \begin{array}{cc} \vdots & 214.52 \end{array} & 262 & \;0\\ \begin{array}{cc} \vdots & 0 \end{array}\; & 0 & 225.24 \end{array} \end{array}\right]=\left[\begin{array}{cc} A_{S} & B_{T}\\ B_{T} & D_{S} \end{array}\right]$$

For the first strip of the plate, the E-T and S-B couplings were recognized as in Equation (13):

(13) $$\left[\begin{array}{cc} \begin{array}{ccc} 73968.63 & 11830.44 & -3633.85\\ 11830.44 & 15827.10 & -3633.85\\ \begin{array}{c} -3633.85\\ \cdots \end{array} & \begin{array}{c} -3633.85\\ \cdots \end{array} & \begin{array}{c} 13106.80\\ \cdots \end{array} \end{array} & \begin{array}{ccc} \begin{array}{cc} \vdots & -4491.73 \end{array} & 1439.30 & -1526.22\\ \begin{array}{cc} \vdots & \;1439.30 \end{array} & 1613.13 & -1526.22\\ \begin{array}{c} \begin{array}{cc} \vdots & -1526.22 \end{array}\\ \begin{array}{cc} \cdots & \;\cdots \end{array}\; \end{array} & \begin{array}{c} -1526.22\\ \cdots \end{array} & \begin{array}{c} 1439.30\\ \cdots \end{array} \end{array}\\ symmetry & \begin{array}{ccc} \begin{array}{cc} \vdots & \;3565.79 \end{array}\; & 457.03 & -403.97\\ \;\begin{array}{cc} \vdots & \;457.03 \end{array}\; & 3565.79 & -403.97\\ \begin{array}{cc} \vdots & -403.97 \end{array}\; & 403.97 & 514.49 \end{array} \end{array}\right]=\left[\begin{array}{cc} A_{F} & B_{F}\\ B_{F} & D_{F} \end{array}\right]$$

The ABD matrix for the second strip of the plate representing E-T and S-B couplings is given in Equation (14):

(14) $$\left[\begin{array}{cc} \begin{array}{ccc} 15827.10 & 11830.44 & 3633.85\\ 11830.44 & 73968.64 & 3633.85\\ \begin{array}{c} 3633.85\\ \cdots \end{array} & \begin{array}{c} 3633.85\\ \cdots \end{array} & \begin{array}{c} 13106.80\\ \cdots \end{array} \end{array} & \begin{array}{ccc} \begin{array}{cc} \vdots & -1613.13 \end{array} & -1439.30 & -1526.22\\ \begin{array}{cc} \vdots & -1439.30 \end{array} & \;4491.73 & -1526.22\\ \begin{array}{c} \begin{array}{cc} \vdots & -1526.22 \end{array}\\ \begin{array}{cc} \cdots & \;\cdots \end{array}\; \end{array} & \begin{array}{c} -1526.22\\ \cdots \end{array} & \begin{array}{c} -1439.30\\ \cdots \end{array} \end{array}\\ symmetry & \begin{array}{ccc} \begin{array}{cc} \vdots & \;627.83\; \end{array} & \;457.03 & \;403.97\\ \begin{array}{cc} \vdots & \;457.03\; \end{array}\; & \;3565.79\; & \;403.97\\ \begin{array}{cc} \vdots & \;403.97\; \end{array} & \;403.97\; & \;514.49 \end{array} \end{array}\right]=\left[\begin{array}{cc} A_{F} & B_{F}\\ B_{F} & D_{F} \end{array}\right]$$

The ABD matrix for the final configuration representing E-B, E-T, and S-B couplings is given in Equation (15):

(15) $$\left[\begin{array}{cc} \begin{array}{ccc} 107618.73 & 38254.07 & 0\\ 38254.07 & 107618.73 & 0\\ \begin{array}{c} 0\\ \cdots \end{array} & \begin{array}{c} 0\\ \cdots \end{array} & \begin{array}{c} 41536.15\\ \cdots \end{array} \end{array} & \begin{array}{ccc} \begin{array}{cc} \vdots & -39681.60 \end{array}\; & 0 & 381.55\\ \begin{array}{cc} \vdots \; & 0 \end{array}\; & 39681.60 & 381.55\\ \begin{array}{c} \begin{array}{cc} \vdots & \;381.55 \end{array}\;\\ \;\begin{array}{cc} \cdots & \;\cdots \end{array}\; \end{array} & \begin{array}{c} 381.55\\ \cdots \end{array} & \begin{array}{c} 0\\ \cdots \end{array} \end{array}\\ symmetry & \begin{array}{ccc} \begin{array}{cc} \vdots & \;37727.85 \end{array} & 5694.84 & \;0\\ \begin{array}{cc} \vdots \; & 5694.84 \end{array} & 37727.85 & \;0\\ \begin{array}{cc} \vdots \; & 0 \end{array}\; & 0 & 6671.83 \end{array} \end{array}\right]=\left[\begin{array}{cc} A_{S} & B_{LT}\\ B_{LT} & D_{S} \end{array}\right]$$

Analytical calculations confirm the presence of B-T and E-T matrix couplings for the chosen configuration in each plate element region.

## 5. Experimental Tests

Plate elements with the configuration presented in Table 1 were manufactured for three angles of fiber alignments: 30°, 45°, and 60°, with constant thickness (single layer thickness was 0.105 mm). Carbon fiber/epoxy (EP137-CR527/100-35) material was prepared using a standard autoclave curing process. The laminate manufacturing process was carried out under special and sterile conditions [30].

**Figure 2:**
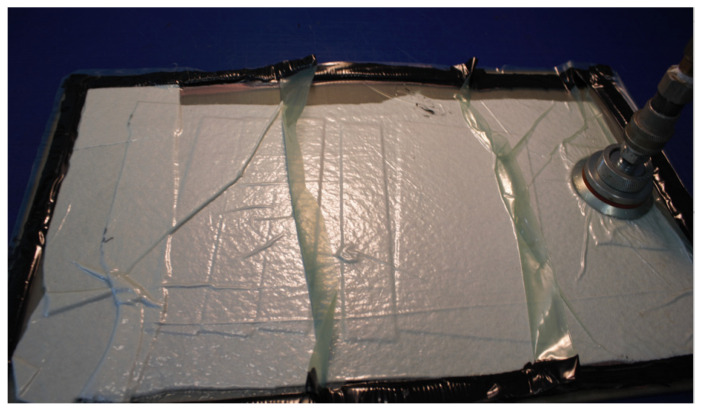
Manufactured process—vacuum package.

The manufacturing process included the preparation of a hermetic vacuum package in a special air-conditioned “clean room”, on a prepared form, enabling the mapping of the dimensions and shape of the manufactured elements (Figure 2). After the autoclave process, the central cut-out was made by a milling process using a special kind of milling cutter.

**Figure 3:**
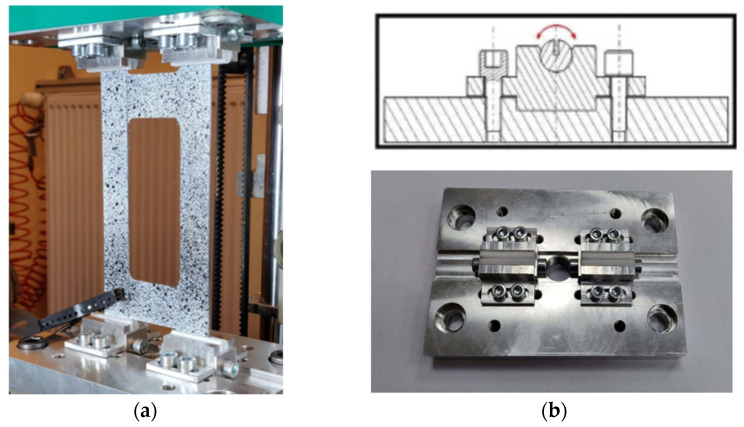
(**a**) Universal testing machine illustrating the plate element under test; (**b**) specially designed grips.

The samples were rectangular plates with constant overall dimensions of 120 × 80 mm weakened by central cut-outs with variable geometric parameters *a* and *b* and with the constant corners’ rounded radius equal to 5 mm. On the shorter edges of the plate above and below the central cut-out, there are technological recesses with a width equal to the width of cut-out b, with the same radius as the cut-out corners. The task of the technological recesses was the constructional separation of zones, where only twisting and bending occur, without the effects of boundary conditions related to the fastening. Experimental validation of E-T and E-B couplings’ influence on plate behavior was performed using the Instron universal testing machine modernized by Zwick-Roell and equipped with specially designed grips (Figure 3). The experimental tests were performed at a constant velocity of the cross-bar equal to 2 mm/min and in ambient temperature. During the tests, force loading, plate displacement, and plate deflection in the perpendicular direction to the vertical strips of the plate in the middle of the height of the strip were measured. More details about experimental tests and results are given in previous articles [31,32].

## 6. FEM Method

**Figure 4:**
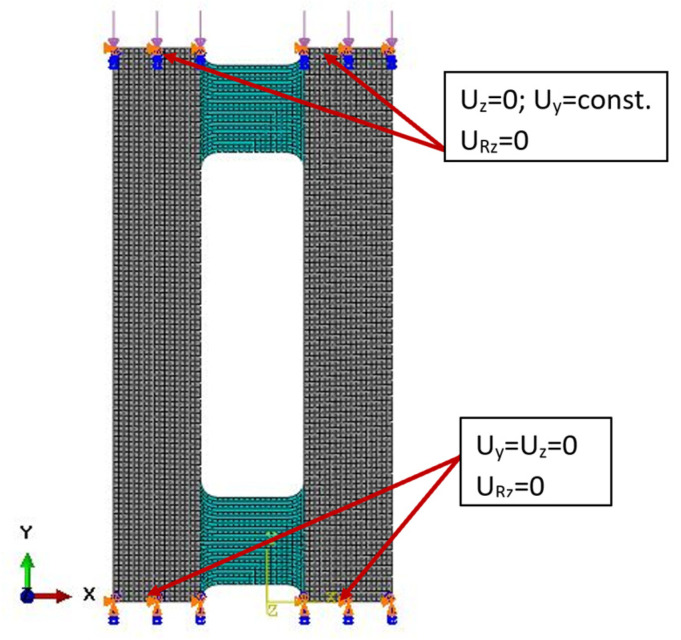
Numerical model with boundary conditions.

To predict the extension–twisting and extension–bending couplings, an Abaqus analysis with FEM was used for comparison with the experimental tests using four-node shell elements (S4R) with quadratic shape function and reduced integration. The discrete numerical model consisted of 4166 finite elements. The plate was simply supported and loaded by axial load along the upper edge. The numerical model with boundary conditions is presented in Figure 4. 

**Figure 5:**
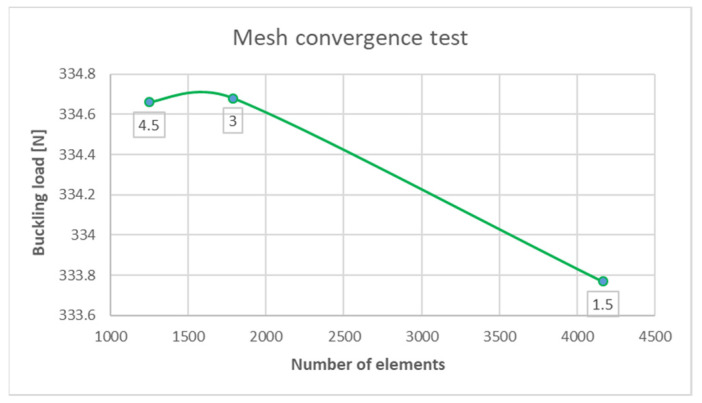
The convergence test.

Moreover, to check the effect of meshing size, three different element sizes were tested: 1.5 mm, 3 mm, and 4.5 mm. The mesh convergence was established by increasing the mesh density in the plate model. It was observed that there were no considerable changes in load response between 1.5 mm and 4.5 mm element size (Figure 5). Therefore, an element size of 1.5 mm was used in subsequent analysis. It was confirmed also by [33,34], that the size of the FEM mesh is of negligible importance in the FEM method.

More details about the numerical analysis are presented in [35,36].

## 7. Results and Discussion

**Figure 6:**
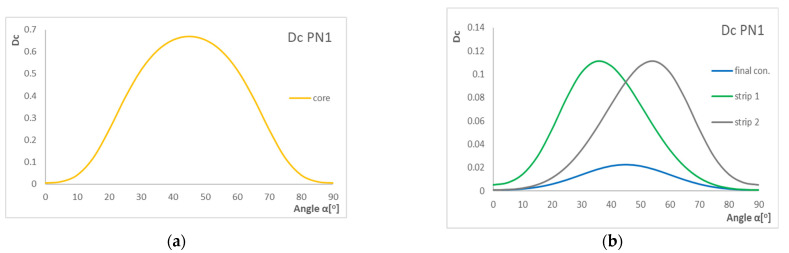
Influence of layer angle α in laminate with PN1 stacking sequence on D_c_ coefficient (**a**) for core and (**b**) for strips and final configuration.

**Figure 7:**
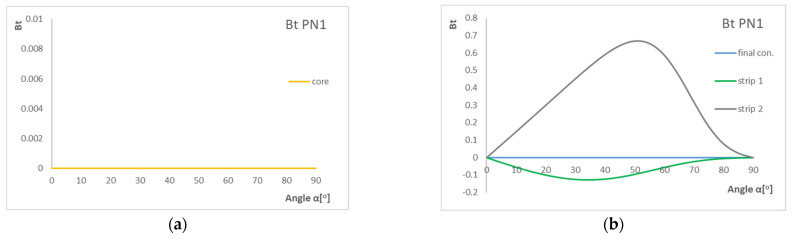
Influence of layer angle α in laminate with PN1 stacking sequence on B_t_ coefficient (**a**) for core and (**b**) for strips and final configuration.

**Figure 8:**
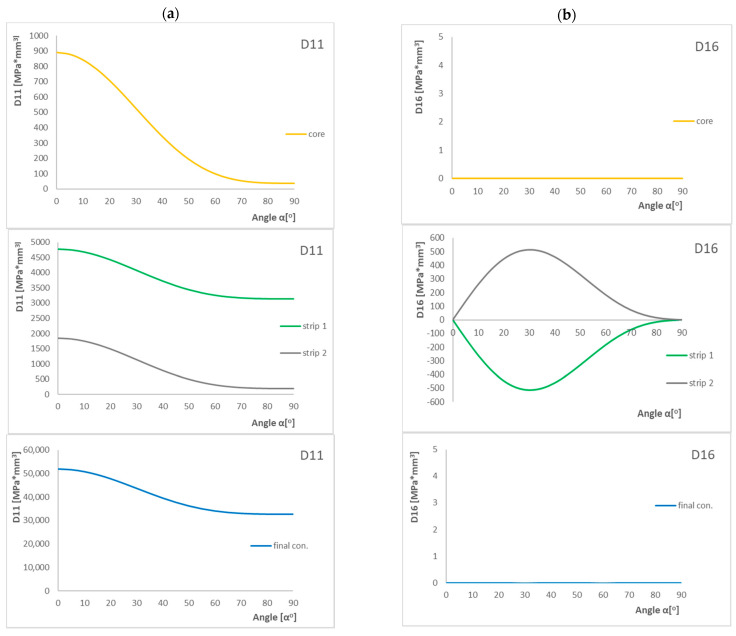

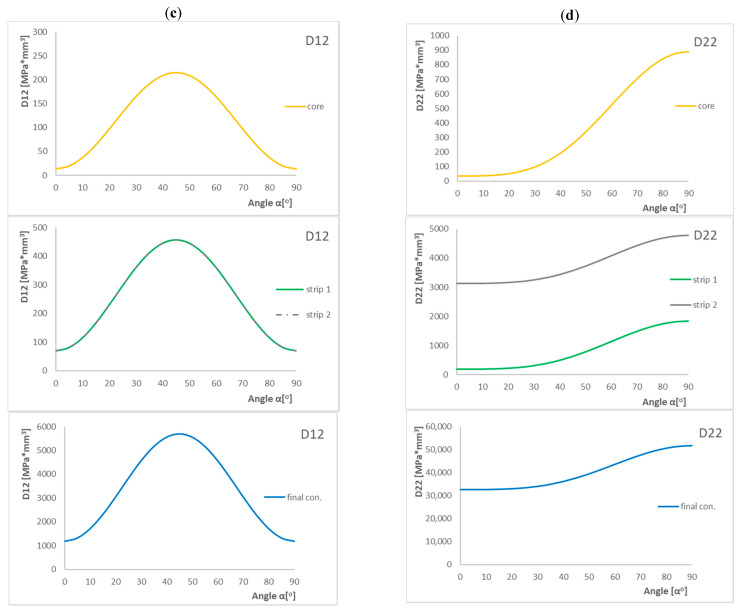
Influence of layer angle α in laminate with PN1 stacking sequence on the (**a**) D_11_, (**b**) D_16_, (**c**) D_12_, and (**d**) D_22_ components.

Figure 6, Figure 7 and Figure 8 present the results of the *D*_c_ coefficient. The graphs show the influence of laying layer angle α in laminates on the *D*_c_ coefficient value. For the tested configuration, we made graphs for the core, strip(s), and final configuration.

As we can see, the highest value of the *D*_c_ parameter is for angles around 30–50°. Furthermore, the *D*_c_ parameter is the highest for the core. Moreover, we can observe some displacement between strip 1 and strip 2, but this movement is a result of layers 0° and 90° added at the end of the configuration. For the laminates rich in 0° and 90° plies, the *D*_c_ is always small.

Figure 7 presents the results of the *B*_t_ coefficient. The graphs show the influence of laying layer angle α in laminate with three configurations on the *B*_t_ coefficient value.

Based on the graphs, the highest value of the *B*_t_ parameter is for angles around 40–60°, and the highest B-T coupling intensity is for strips. For the core, parameter *B*_t_ equals zero, which means that there is no influence of B-T coupling. For strips, we can observe that the graphs are not symmetric in relation to the horizontal axis. For angles 0° and 90°, the laminate becomes a ply with no B-T coupling intensity.

Figure 8 presents the results of components of *D*_c_ and *B*_t_ parameters and the influence of laying layer angle α on their value.

Analyzing the above cases, we can observe that the D_11_ component decreases from 0° to 90°, and the highest decrease is for the core. The opposite is true for the D_22_ component—as the angle increases, the value of the coefficient also increases. The next component which influences the *D*_c_ coefficient is D_12_, and the behavior of this component changes parabolically. The highest value is at 45°. The last component which together with the D_11_ component has an influence on the *B*_t_ coefficient is D_16_, and the graph of the influence of layer angle α on the value of this component looks like a parabola but with the maximum shifted to the left side. Only for the core, D_16_ does not change and is zero for all angles. However, it should also be noted that the situation is the same for the final configuration of the PN1 arrangement of layers. 

**Figure 9:**
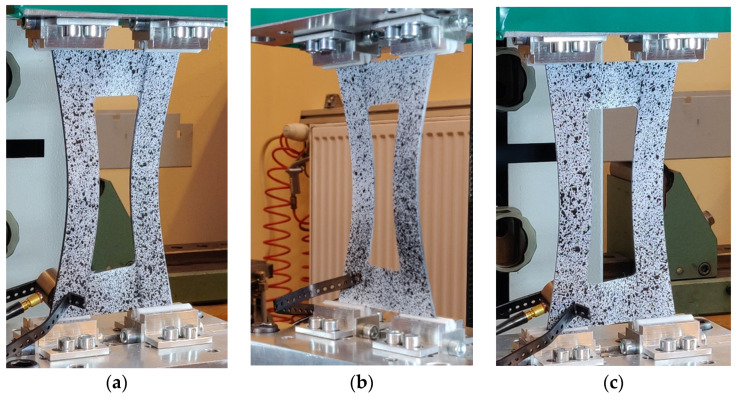
Experimental results for plate with angles (**a**) 30°, (**b**) 45°, and (**c**) 60°.

**Figure 10:**
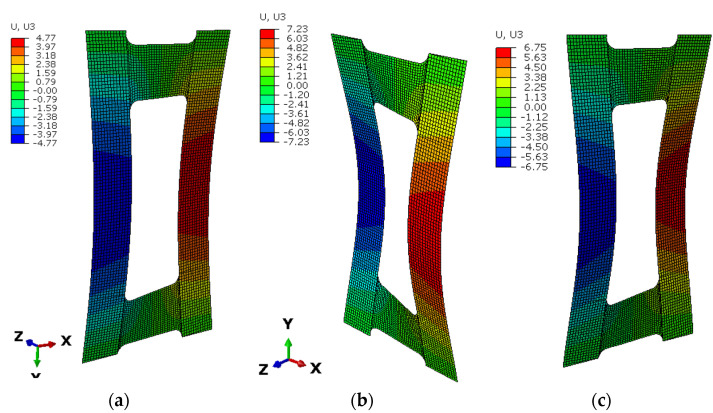
Numerical results of deflection for plate with angles (**a**) 30°, (**b**) 45°, and (**c**) 60°.

Figure 9 presents results from experimental tests which confirmed that the appropriate selection of asymmetric configuration and matrix couplings can bring the expected results. For all tested angles, the stable flexural–torsional form was obtained. Figure 10 presents the deflection results from numerical analysis, where the differences for three tested angles can be observed. Nonlinear analysis was performed according to the Tsai–Wu criterion. The results confirm the above analytical results for the *Bt* parameter, for which the highest value was for angles around 40–60° and the highest B-T coupling intensity was for strips.

## 8. Conclusions

This paper presents a study of elastic matrix couplings in asymmetrical configurations which were used to obtain the elastic element. The study focused on the ply sequences expected to have the strongest effect on obtaining the flexural–torsional buckling mode as the naturally lowest mode of buckling of a plate element. Thanks to this, the plate element can work as an elastic element. For this reason, extension–twisting (E-T) and extension–bending (E-B) coupled laminate classes were chosen for consideration. The analysis of laminate behavior associated with elastic couplings was performed according to the Classical Laminate Theory. 

Additional analysis of the influence of the laying fiber angle on the D_11_, D_12_, D_16_, and D_22_ components of the bending stiffness matrices showed that the angle of laying fibers around 40–50° significantly amplifies the effects of elastic couplings. In the case of laminates rich in 0° and 90° plies, the *D*_c_ is always small. The highest value of the *B*_t_ parameter is for angles around 40–50° and the highest B-T coupling intensity is for strips. This means that for this range of angles, we can obtain a plate element that can transfer the highest loads. In the case of 0° and 90°, the laminate becomes a ply with no B-T coupling intensity. 

The main aim of the work, which was to show that it is possible to design a specimen with maximized B-T coupling and to determine how we can influence this coupling intensity, was achieved.

The results obtained from this study conducted on real structures provide new and important information regarding the design and possibilities of using a thin-walled plate element as an elastic element. The results also indicate an area of research that is worth further analysis, which is how, by the appropriate selection of matrix components, we can influence a structure’s behavior.

## Figures and Tables

**Table 1:** Tested laminate configuration.

Plies No.	Core		Strips		Final Configuration	Symbol
	Ply Orientations	Couplings	Ply Orientations	Couplings		
18	[α/−α]_2_	A_S_B_T_D_S_ *	[α/−α/0/−α/0/α/90/α/−α]	A_S_B_L_D_S_ **	[0_3_/α/−α/0/−α/α/−α/α/−α/α/90/α/−α/90_3_]_T_	PN1

“α” is the angle of ply orientation. * The ply orientation given by York [12,27], consisting of 2 plies only; to meet the assumptions of the study, the core of the investigated plate consisted of 4 plies. ** The ply orientation proposed by York was modified for the purpose of this study—in the final stacking sequence, the “0″ ply was replaced with [α/−α]2 plies (plate core); additionally, triple “0” and “90” plies were added in the vertical strips.

## Data Availability

Not applicable.
